# Epidemiology and Clinical Features of Respiratory Viruses in Hospitalized Iranian Children During the COVID‐19 Pandemic

**DOI:** 10.1002/iid3.70275

**Published:** 2025-09-29

**Authors:** Ali Nateghi, Morvarid Hamrahjoo, Mohammad Yasaghi, Mahnaz Ramzali, Saeed Samadizadeh, Fatemeh Fotouhi, Vahid Salimi, Lobat Shahkar, Britt Nakstad, Alireza Tahamtan

**Affiliations:** ^1^ Department of Microbiology, Faculty of Medicine Golestan University of Medical Sciences Gorgan Iran; ^2^ Wellcome‐Wolfson Institute for Experimental Medicine Queen's University Belfast Belfast Northern Ireland UK; ^3^ Influenza and Other Respiratory Viruses Pasteur Institute of Iran Tehran Iran; ^4^ Department of Virology Tehran University of Medical Sciences Tehran Iran; ^5^ Department of Pediatrics, Faculty of Medicine Golestan University of Medical Sciences Gorgan Iran; ^6^ Department of Pediatric and Adolescent Health University of Botswana Gaborone Botswana; ^7^ Division of Paediatric and Adolescent Medicine, Institute of Clinical Medicine University of Oslo Oslo Norway; ^8^ Infectious Diseases Research Center Golestan University of Medical Sciences Gorgan Iran

**Keywords:** acute respiratory tract infection, children, COVID‐19, epidemiology, respiratory viruses

## Abstract

**Background:**

Acute respiratory infections (ARIs) are a significant global health concern, especially in children under five, causing approximately 4.3 million annual deaths. ARIs are mainly caused by respiratory viruses. The coronavirus disease 2019 (COVID‐19) has altered the circulation of respiratory viruses. This study investigates the epidemiology and clinical features of respiratory viruses in hospitalized children during the COVID‐19 pandemic in Gorgan, Iran.

**Methods:**

A total of 264 nasopharyngeal swab samples were collected from hospitalized children between October 2021 to March 2022 at Taleghani Children's Hospital, Gorgan, Iran. The frequency of various respiratory viruses, including human parainfluenza viruses (HPIV1‐4), influenza viruses A and B (FLU‐A, B), human metapneumovirus (HMPV), human rhinovirus (HRV), respiratory syncytial virus (RSV), and severe acute respiratory syndrome‐associated coronavirus 2 (SARS‐CoV‐2) was detected using a SYBR green‐based real‐time PCR assay.

**Results:**

Out of the 264 hospitalized children, 88.2% (233) tested positive for at least one respiratory virus, with 60.2% (159) showing co‐infections and 28% (74) having single infections. The most frequently detected were HRV (56.4%), HMPV (53%), and RSV (18.2%). The proportions of HPIV‐1, HPIV‐2, HPIV‐3, HPIV‐4, FLU‐A, FLU‐B, and SARS‐CoV‐2 were 8.7%, 12.9%, 8%, 7.6%, 1.9%, 0%, and 15.2%, respectively. There was a clear association between specific viruses and some clinical symptoms, such as RSV with pneumonia, and HPIV‐1 with cyanosis. Co‐infections were linked to severe outcomes, including pneumonia and seizures. Among all 264 patients, 5 died, and 3 of them had underlying diseases. All fatal cases tested positive for at least one virus, with HMPV being the most frequently detected.

**Conclusions:**

This study highlights the considerable impact of ARIs among children under five in Golestan Province, Iran, during the COVID‐19 pandemic. The findings underscore the importance of early detection and ongoing surveillance, particularly in high‐risk pediatric populations and across diverse geographic areas.

**Abbreviations**
ARIs: acute respiratory infections
BLAST: basic Local Alignment Search Tool
cDNA: complementary DNA
COVID‐19: coronavirus disease‐2019
FLU‐A: influenza viruses A
FLU‐B: influenza viruses B
HCoVs: human coronaviruses
HMPV: human metapneumovirus
HN: hemagglutinin‐neuraminidase
HPIVs: human parainfluenza viruses
HRV: human rhinovirus
ICU: intensive care unit
LRTIs: lower respiratory tract infections
NCBI: National Center for Biotechnology Information
RSV: respiratory syncytial virus
SARS‐CoV‐2: severe acute respiratory syndrome‐associated coronavirus 2

## 1 Introduction

Acute respiratory infections (ARIs) cause approximately 4.3 million pediatric deaths every year and pose a significant global health burden, particularly among children under the age of five [1, 2]. The etiology is complex and diverse, with variations depending on gender, age, and season [3]. These infections encompass a wide range of illnesses, from upper respiratory tract infections, rhinopharyngitis, and sinusitis to more severe lower respiratory tract infections (LRTIs), such as pneumonia and bronchiolitis [4]. Young children are particularly vulnerable to the harmful effects of ARIs due to their immature immune systems and limited physiological reserves. The severity of respiratory symptoms remains a significant health challenge, incurring substantial economic and social burdens. This underscores the need for a deeper understanding of the epidemiology and the various factors that trigger these symptoms [5].

Among the various etiological agents responsible for ARIs, viruses are the predominant cause of childhood respiratory infections and disease [6]. In children with community‐acquired pneumonia, more than two‐thirds of cases are caused by respiratory viruses [7]. The most prevalent viruses linked to ARIs are human parainfluenza viruses (HPIVs), influenza viruses A and B (FLU‐A, B), human metapneumovirus (HMPV), human rhinovirus (HRV), respiratory syncytial virus (RSV), and human coronaviruses (HCoVs) [8]. Infection with respiratory viruses, whether occurring individually or as co‐infections, is frequently observed in children and has been associated with a wide range of clinical outcomes, including increased disease severity and more complex clinical management [9]. Respiratory viruses are widespread and can cause a range of clinical symptoms and manifestations, including life‐threatening illnesses, particularly in high‐risk groups such as children, older adults, and individuals with underlying health conditions. Accurate detection of the specific virus can provide information on appropriate infection control measures and the prescription of suitable antiviral drugs [10, 11]. This could potentially reduce prolonged hospital stays and allow for the discontinuation of unnecessary antibiotics [12, 13].

Following the emergence of the novel coronavirus disease 2019 (COVID‐19) pandemic, the epidemiology and transmission patterns of respiratory viruses have shifted, although our understanding remains limited. The measures implemented to prevent severe acute respiratory syndrome‐coronavirus 2 (SARS‐CoV‐2) infection likely played a role in this change [14]. Given the varying reports on the circulation of respiratory viruses during the COVID‐19 pandemic, and the limited data on the prevalence and clinical impact of viral co‐infections in children, we aimed to investigate the epidemiology and clinical characteristics of respiratory viral infections in infants and children in Golestan Province, located in northern Iran.

## 2 Materials and Methods

### 2.1 Study Participants

A total of 264 samples were collected from hospitalized children between October 2021 and March 2022. All children suspected of having a viral ARI were included in the study. The inclusion criteria were being under 5 years old and presenting symptoms indicative of virus‐induced ARIs, such as coughing and fever. The exclusion criteria included patients with missing or incomplete clinical or demographic data required for the study, as well as samples that were not stored or transported under appropriate conditions. The children were hospitalized at the only specialized pediatric center in the Golestan Province, Taleghani Children's Hospital, Gorgan, Iran. The samples were collected during the COVID‐19 pandemic and transported in viral transport media at 4°C to the Microbiology department of Golestan University of Medical Sciences and tested for SARS‐CoV‐2 and RSV. Demographic and clinical information of the patients was collected using a questionnaire completed by their caregivers and relatives. The parents of all participants were informed about the purpose of the study, and their informed consent was obtained. This study was approved by the Science and Bioethics Committee of Golestan University of Medical Sciences (IR.GOUMS.REC.1401.528 ‐ IR.GOUMS.REC.1401.527).

### 2.2 RNA Extraction and Positive Control Preparation

**Figure 1:**
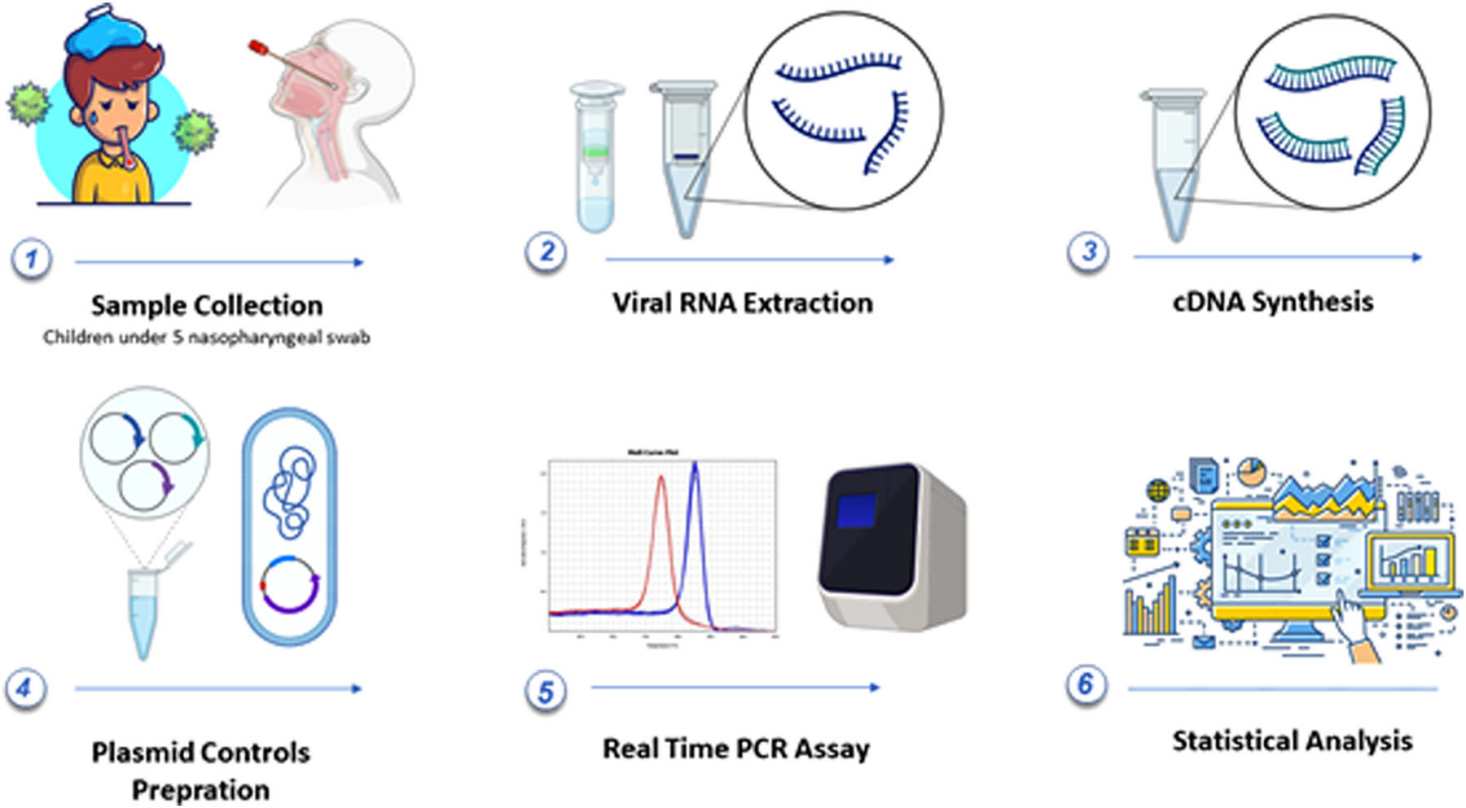
Flowchart showing the procedure in the present study.

The study flowchart is shown in Figure 1. Briefly, 200 µL of all samples was used for extraction from nasopharyngeal swabs using a commercial high‐purity Viral RNA Extraction Kit (BehPrep, Iran), following the manufacturer's instructions. The quantity and quality of the extracted samples were assessed using a spectrophotometer (DeNovix, USA). All samples were validated for verification of extraction using a specific primer for the Glyceraldehyde‐3‐phosphate dehydrogenase (GAPDH) gene. The extracted RNA was converted into complementary DNA (cDNA) using the cDNA reverse transcription kit (Yekta Tajhiz Azma, Iran) according to the manufacturer's instructions.

Positive controls for HPIV (types 1–4) were provided by the Pasteur Institute of Iran, in the form of constructed plasmids containing the full‐length hemagglutinin‐neuraminidase (HN) gene [15]. To construct plasmids for positive controls, other than SARS‐CoV‐2 and RSV, the genomic sequences of the target gene of FLU‐A (M gene), FLU‐B (M gene), HMPV (M gene), and HRV (5′‐NC region gene) were amplified and inserted into the TA vector (pUCM‐T Cloning Vector Kit, Bio basic, Canada) with meticulous attention to detail. The plasmids were transformed into competent *Escherichia coli* TOP10F and purified according to the standard protocols.

### 2.3 Real‐Time PCR Assay

Presence of HPIV (types 1–4) (HN gene), FLU‐A (M gene), FLU‐B (M gene), HMPV (M gene), HRV (5′‐NC region gene) genomes in collected samples were analyzed through the SYBR green‐based real‐time PCR assay using specifically designed primers for each target gene of viruses (Supporting Information S1: Table S1). Primer specificity was confirmed by performing in silico analysis using Basic Local Alignment Search Tool (BLAST) against the National Center for Biotechnology Information (NCBI) nucleotide database to ensure target‐specific amplification and the absence of cross‐reactivity. Briefly, 100 ng of synthesized cDNA was added to the total volume of 20 µL reaction mixture containing: Master Mix (RealQ Plus 2x Master Mix Green, Ampliqon, Denmark), distilled water, and forward and reverse primers. The reaction conditions were: 95°C for 15 min followed by 40 cycles of 95°C for 20 s, 58°C for 30 s and 72°C for 30 s. After the last cycle, the temperature increased to 95°C, then decreased to 65°C and slowly increased to 95°C at a rate of 0.1°C per second, with continuous fluorescence monitoring for the melting analysis. The reaction was performed on StepOnePlus ABI instrument (ABI, USA).

### 2.4 Statistical Analysis

Data were collected and analyzed using the SPSS version 26 (IBM, Chicago, IL). Results were presented as the mean and the standard deviation and qualitative data as frequency (percentage). The chi‐square and Fisher's exact tests were used to perform statistical analysis. The chi‐square test was used with a large sample size and expected frequency counts greater than 5, while Fisher's exact test was used when the sample size was small, and the expected frequency counts were less than 5. Significant associations were considered if the *p*‐values were less than 0.05. This study was exploratory in nature, and all findings are interpreted with appropriate caution.

## 3 Results

**Figure 2:**
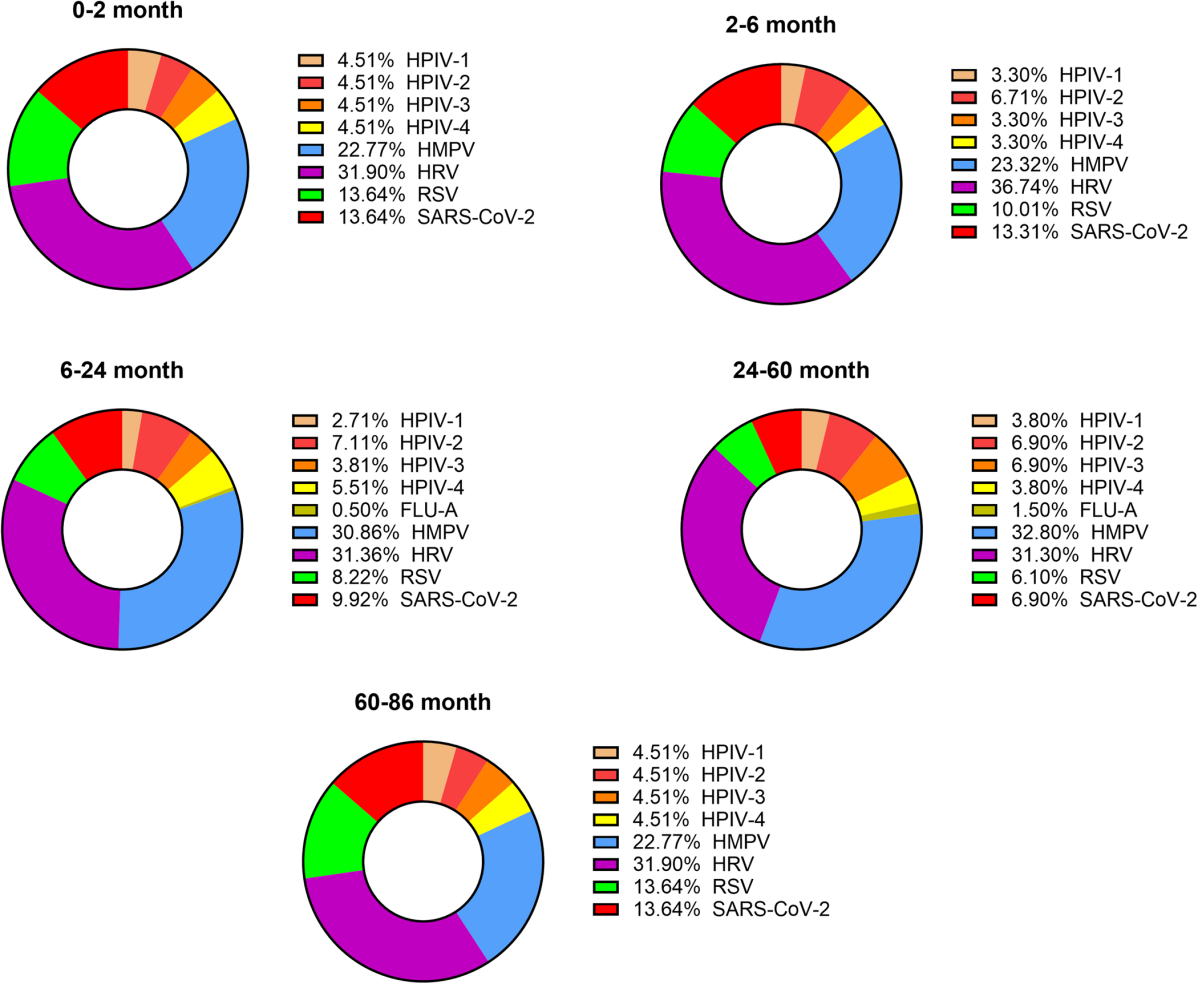
Proportions of viruses detected according to age group (month). The sliced part of each pie graph indicates the virus detected most frequently in that age group.

Among the 264 samples, 173 (65.5%) were male and 91 (34.5%) were female. The children had a high vaccination coverage (86.7%) according to the Iranian routine vaccination schedules under the expanded program on immunization (EPI), appropriate for their age and none were vaccinated for SARS‐CoV‐2. The patients had an average age of 21.2 ± 19.3 months and were categorized into five different age groups. Details of demographic data and distribution of viruses are shown in Table 1. The frequency of HPIV‐1, HPIV‐2, HPIV‐3, HPIV‐4, FLU‐A, FLU‐B, HMPV, HRV, RSV, and SARS‐CoV‐2 were 23 (8.7%), 34 (12.9%), 21 (8%), 20 (7.6%), 5 (1.9%), 0 (0%), 140 (53%), 149 (56.4%), 48 (18.2%), and 40 (15.2%), respectively. HRV was the most frequently detected virus, followed by HMPV and RSV. Although not statistically significant, a trend toward gender disparity was observed in the positive samples. A significant difference was observed between age groups, as well as HPIV‐1 and SARS‐CoV‐2 positive cases (*p* < 0.05). The predominant viruses varied among different age groups, while HRV and HMPV were the most frequent respiratory viruses in children (Figure 2).

**Table 1:** Demographic details and distribution of viruses among patients.

Variable	Cases, *n* (%)	Male, *n* (%)	Female, *n* (%)	*p* value	0–2, *n* (%)	2–6, *n* (%)	6–24, *n* (%)	24–60, *n* (%)	60–86, *n* (%)	*p* value
Total	264 (100)	173 (65.5)	91 (34.5)	—	13 (4.9)	54 (20.5)	106 (40.2)	73 (27.7)	18 (6.8)	—
HPIV‐1	23 (8.7)	14 (5.3)	9 (3.4)	0.62	1 (7.7)	11 (20.4)	5 (4.7)	5 (6.8)	1 (5.6)	0.01
HPIV‐2	34 (12.9)	21 (8)	13 (4.9)	0.62	1 (7.7)	9 (16.7)	13 (12.3)	9 (12.3)	2 (11.1)	0.89
HPIV‐3	21 (8)	11 (4.1)	10 (3.7)	0.18	1 (7.7)	3 (5.6)	7 (6.6)	9 (12.3)	1 (5.6)	0.60
HPIV‐4	20 (7.6)	16 (6)	4 (2.3)	0.15	1 (7.7)	3 (5.6)	10 (9.4)	5 (6.8)	1 (5.6)	0.91
FLU‐A	5 (1.9)	4 (2.3)	1 (1.1)	0.49	0 (0)	2 (3.7)	1 (0.9)	2 (2.7)	0 (0)	0.67
FLU‐B	0 (0)	0 (0)	0 (0)	n/a	0 (0)	0 (0)	0 (0)	0 (0)	0 (0)	n/a
HMPV	140 (53)	94 (35.6)	46 (50.5)	0.55	5 (38.5)	29 (53.7)	56 (52.8)	43 (58.9)	7 (38.9)	0.46
HRV	149 (56.4)	98 (37.1)	51 (19.3)	0.92	7 (53.8)	33 (66.1)	57 (53.8)	41 (56.2)	11 (61.1)	0.91
RSV	48 (18.2)	28 (16.2)	20 (22)	0.24	3 (23.1)	19 (35.2)	15 (14.2)	8 (11)	3 (16.7)	0.00
SARS‐CoV‐2	40 (15.2)	30 (17.3)	10 (11)	0.17	3 (23.1)	6 (11.1)	18 (17)	9 (12.3)	4 (22.2)	0.60

*Note:* The *χ*² test was applied when the sample size was large and the expected frequency counts exceeded 5, whereas Fisher′s exact test was employed for smaller sample sizes with expected counts less than 5.

Abbreviations: FLU‐A, influenza viruses A; FLU‐B, influenza viruses B; HMPV, human metapneumovirus; HPIVs, human parainfluenza viruses; HRV, human rhinovirus; n/a, not applicable due to no positive cases detected for FLU‐B; RSV, respiratory syncytial virus; SARS‐CoV‐2, severe acute respiratory syndrome‐associated coronavirus 2.

Details of clinical data and distribution among positive cases are shown in Table 2. Out of 264 cases, 74 patients (28%) had various underlying conditions, including neuromuscular disease (8.3%), icterus (7.6%), chronic heart disease (5.7%), and congenital heart disease (2.3%). Twenty‐six patients (9.8%) were born prematurely. Common clinical symptoms were cough (81.1%), fever (74.2%), shortness of breath (30.7%), and wheezing (28.4%). Among all, 160 (60.6%), 20 (7.6%), and 8 (3%) cases were diagnosed as pneumonia, bronchiolitis, and asthma, respectively. Additionally, 229 patients (86.7%) were fully vaccinated, while 35 (13.3%) had incomplete vaccination. Oxygen saturation (SpO2) at admission or during hospitalization was over 93% for 83.3% of cases; 14.4% had less than 93% and 39% needed mechanically assisted ventilation. In positive cases, the primary symptoms were cough and fever, along with more severe issues such as difficulty breathing, wheezing, and pneumonia. Statistical analysis revealed significant associations between specific respiratory viruses and clinical manifestations, including: HPIV‐1 with cyanosis; HMPV with cough and pneumonia; HRV with chronic heart disease and loss of consciousness; RSV with pneumonia, wheezing, rapid breathing, and difficulty breathing; and SARS‐CoV‐2 with pneumonia, wheezing, and fever (*p* < 0.05). Furthermore, A significant association was observed between RSV infection and the requirement for ventilator support. All patients received antibiotics, and some received additional treatments such as bronchodilators (183/264, 69.3%), corticosteroids (123/264, 46.6%), oseltamivir (58/264, 22%), epinephrine (25/264, 9.5%), and ribavirin (5/264, 1.9%).

**Table 2:** Relation of positive cases with clinical information of patients.

Characteristics	Total (%)	HPIV‐1 (%)	*p* value	HPIV‐2 (%)	*p* value	HPIV‐3 (%)	*p* value	HPIV‐4 (%)	*p* value	FLU A (%)	*p* value	HMPV (%)	*p* value	HRV (%)	*p* value	RSV (%)	*p* value	SARS‐CoV‐2 (%)	*p* value
**Clinical symptoms**																				
Cough	214 (81.1)	19 (82.6)	1	31 (91.2)	0.10	17 (81)	1	16 (80)	1	4 (80)	1	106 (75.7)	0.01	226 (84.6)	0.09	43 (89.6)	0.08	3 (82.5)	0.80
Fever	196 (74.2)	18 (78.3)	0.64	24 (70.6)	0.60	17 (8.7)	0.46	12 (60)	0.13	5 (100)	0.33	107 (76.4)	0.38	115 (77.2)	0.21	34 (70.8)	0.55	36 (90)	0.01
Difficult breath	81 (30.7)	9 (11.1)	0.35	17 (21)	0.09	8 (38.1)	0.44	7 (35)	0.66	2 (40)	0.64	42 (30)	0.79	44 (29.5)	0.64	22 (45.8)	0.01	9 (22.5)	0.22
Wheezing	75 (28.4)	9 (39.1)	0.23	12 (35.3)	0.34	5 (23.8)	0.62	8 (40)	0.23	3 (60)	0.14	40 (28.6)	0.95	38 (25.5)	0.23	20 (41.7)	0.02	6 (15)	0.04
Loss of consciousness	80 (30.3)	1 (4.3)	1	0 (0)	—	0 (0)	—	0 (0)	—	0 (0)	—	7 (5)	0.95	12 (8.1)	0.00	0 (0)	—	2 (5)	0.98
Vomit	71 (26.9)	8 (34.8)	0.37	9 (26.5)	0.95	9 (42.9)	0.08	5 (25)	0.84	2 (40)	0.61	36 (25.7)	0.64	38 (25.5)	0.56	9 (18.8)	0.15	10 (25)	0.76
Irritability	33 (12.5)	4 (17.4)	0.50	2 (5.9)	0.27	3 (14.3)	0.73	2 (10)	1	2 (40)	0.11	19 (13.6)	0.57	23 (15.4)	0.10	6 (12.5)	1	8 (20)	0.11
Fast breath	25 (9.5)	2 (8.7)	1	5 (14.7)	0.34	0 (0)	—	3 (15)	0.41	0 (0)	—	15 (10.7)	0.46	10 (6.7)	0.08	9 (18.8)	0.01	2 (5)	0.29
Cyanosis	12 (4.5)	4 (17.4)	0.01	1 (2.9)	1	0 (0)	—	2 (10)	0.22	0 (0)	—	6 (4.3)	0.83	6 (4)	0.46	4 (8.3)	0.34	0 (0)	—
Chest pain	2 (0.8)	0 (0)	—	0 (0)	—	0 (0)	—	0 (0)	—	0 (0)	—	2 (1.4)	0.50	1 (0.7)	0.51	0 (0)	—	0 (0)	0.76
**Underlying diseases**																				
Neuromuscular disease	22 (8.3)	3 (13)	0.42	1 (2.9)	0.32	2 (9.5)	0.69	2 (10)	0.67	0 (0)	—	13 (9.3)	0.55	15 (10.1)	0.24	4 (8.3)	1	3 (7.5)	1
Icterus	20 (7.6)	3 (13)	0.39	4 (11.8)	0.30	2 (9.5)	0.66	1 (5)	1	1 (20)	0.32	10 (7.1)	0.77	12 (8.1)	0.73	3 (6.3)	1	4 (10)	0.52
Chronic heart disease	15 (5.7)	1 (4.3)	1	4 (11.8)	0.11	3 (14.3)	0.10	3 (15)	0.09	0 (0)	—	8 (5.7)	0.98	4 (2.7)	0.01	4 (8.3)	0.38	3 (7.5)	0.59
Congenital heart disease	6 (2.3)	0 (0)	—	0 (0)	—	0 (0)	—	1 (5)	0.35	0 (0)	—	4 (2.9)	0.68	4 (2.7)	0.70	0 (0)	—	0 (0)	0.29
Blood disease	3 (1.1)	1 (0.8)	0.24	0 (0)	—	0 (0)	—	0 (0)	—	0 (0)	—	2 (0.7)	0.58	1 (0.7)	0.49	1 (2.1)	0.45	1 (2.5)	0.39
Kidney disease	3 (1.1)	0 (0)	—	0 (0)	—	0 (0)	—	0 (0)	—	0 (0)	—	2 (1.4)	1	2 (1.3)	1	1 (2.1)	0.45	1 (2.5)	0.39
Genetic disease	2 (0.8)	0 (0)	—	0 (0)	—	0 (0)	—	0 (0)	—	0 (0)	—	1 (0.7)	1	0 (0)	—	0 (0)	— (0)	0 (0)	—
UTI	2 (0.8)	0 (0)	—	0 (0)	—	0 (0)	—	0 (0)	—	0 (0)	—	0 (0)	—	1 (0.9)	1	0 (0)	—	1 (2.5)	0.28
Down syndrome	1 (0.4)	0 (0)	—	0 (0)	—	0 (0)	—	0 (0)	—	0 (0)	—	1 (0.7)	1	1 (0.7)	1	0 (0)	—	0 (0)	—
Premature	26 (9.8)	2 (8.7)	0.93	6 (17.6)	0.24	1 (4.8)	0.70	2 (10)	1	0 (0)	—	14 (10)	0.93	17 (11.4)	0.33	4 (8.3)	0.82	3 (7.5)	0.78
**Respiratory involvement**																				
Pneumonia	160 (60.6)	16 (69.6)	0.35	25 (73.5)	0.09	12 (57.1)	0.73	10 (50)	0.31	2 (40)	0.38	77 (55)	0.04	83 (55.7)	0.06	37 (77.1)	0.01	17 (42.5)	0.01
Bronchiolitis	20 (7.6)	2 (8.7)	0.68	4 (11.8)	0.30	4 (19)	0.06	3 (15)	0.18	0 (0)	—	11 (7.9)	0.85	11 (7.4)	0.89	4 (8.3)	0.82	1 (2.5)	0.18
Asthma	8 (3)	1 (4.3)	0.52	1 (2.9)	1	1 (4.8)	0.49	0 (0)	—	0 (0)	—	3 (2)	0.40	3 (2)	0.30	1 (2.1)	0.67	0 (0)	—
**Vaccination**																				
Complete vaccination	229 (86.7)	19 (82.6)	0.52	27 (79.4)	0.18	19 (90.5)	1	19 (95)	0.48	4 (80)	0.51	125 (89.3)	0.19	129 (86.3)	0.92	37 (77.1)	0.02	35 (87.5)	0.87
Incomplete vaccination	35 (13.3)	4 (17.4)		7 (20.6)		2 (9.5)		1 (5)		1 (20)		15 (10.7)		20 (13.4)		11 (22.9)		5 (12.5)	
**SPO_2_**																				
> 93%	220 (83.3)	19 (82.6)	0.69	27 (79.4)	0.36	19 (90.5)	0.59	15 (75)	0.30	5 (100)	0.60	117 (83.6)	0.25	122 (81.9)	0.23	35 (72.9)	0.09	35 (87.5)	0.69
< 93%	38 (14.4)	4 (17.4)		7 (20.6)		2 (9.5)		5 (25)		0 (0)		18 (12.9)		24 (16.1)		11 (22.9)		4 (10)	
Unknown	6 (2.3)	0 (0)		0 (0)		0 (0)		0 (0)		0 (0)		5 (3.6)		3 (2)		2 (4.2)		1 (2.5)	
**Medical support**																				
Ventilation	103 (39)	10 (43.5)	0.64	17 (50)	0.15	11 (52.4)	0.19	10 (50)	0.29	1 (20)	0.65	48 (34.3)	0.09	55 (36.9)	0.42	30 (62.5)	0.00	14 (35)	0.57
**Disease severity**																				
ICU	19 (7.2)	2 (8.7)	0.67	5 (14.7)	0.08	2 (9.5)	0.65	3 (15)	0.16	0 (0)	—	10 (7.1)	0.97	9 (6)	0.40	2 (4.2)	0.54	3 (7.5)	1
**The outcome of the disease**																				
Death	5 (1.9)	1 (4.3)	0.36	1 (2.9)	0.50	0 (0)	—	0 (0)	—	0 (0)	—	4 (2.9)	0.37	3 (2)	1	0 (0)	—	1 (2.5)	0.56

*Note:* The *χ*² test was applied when the sample size was large and the expected frequency counts exceeded 5, whereas Fisher′s exact test was employed for smaller sample sizes with expected counts less than 5.

Abbreviations: FLU‐A, influenza viruses A; HMPV, human metapneumovirus; HPIVs, human parainfluenza viruses; HRV, human rhinovirus; ICU, intensive care unit; RSV, respiratory syncytial virus; SARS‐CoV‐2, severe acute respiratory syndrome‐associated coronavirus 2; SPO_2_, oxygen saturation; UTI, urinary tract infection.

Among all cases, 7.2% were hospitalized in the intensive care unit (ICU), and five cases (1.9%) resulted in death, but there was no significant association with the severity of symptoms. Notably, all five deceased cases tested positive for at least one virus, with HMPV being the most frequently detected virus, in all cases. Co‐infections were identified involving HMPV with HPIV‐1, HPIV‐2, and HRV. The first case exhibited a triple viral co‐infection with HMPV, HRV, and HPIV‐1. The second case presented with a co‐infection involving HMPV and HRV, and the third case with HMPV and HPIV‐2. The remaining two cases were detected as only HMPV infection. All cases presented with severe symptoms, including fever, cough, bronchiolitis, and pneumonia, requiring ventilator support (Table 2). They were all provided with empirical antibiotic therapy initiated at admission in accordance with local hospital protocols, and some received additional treatments such as bronchodilators (4/5), corticosteroids (4/5), oseltamivir (2/5), and epinephrine (1/5).

**Figure 3:**
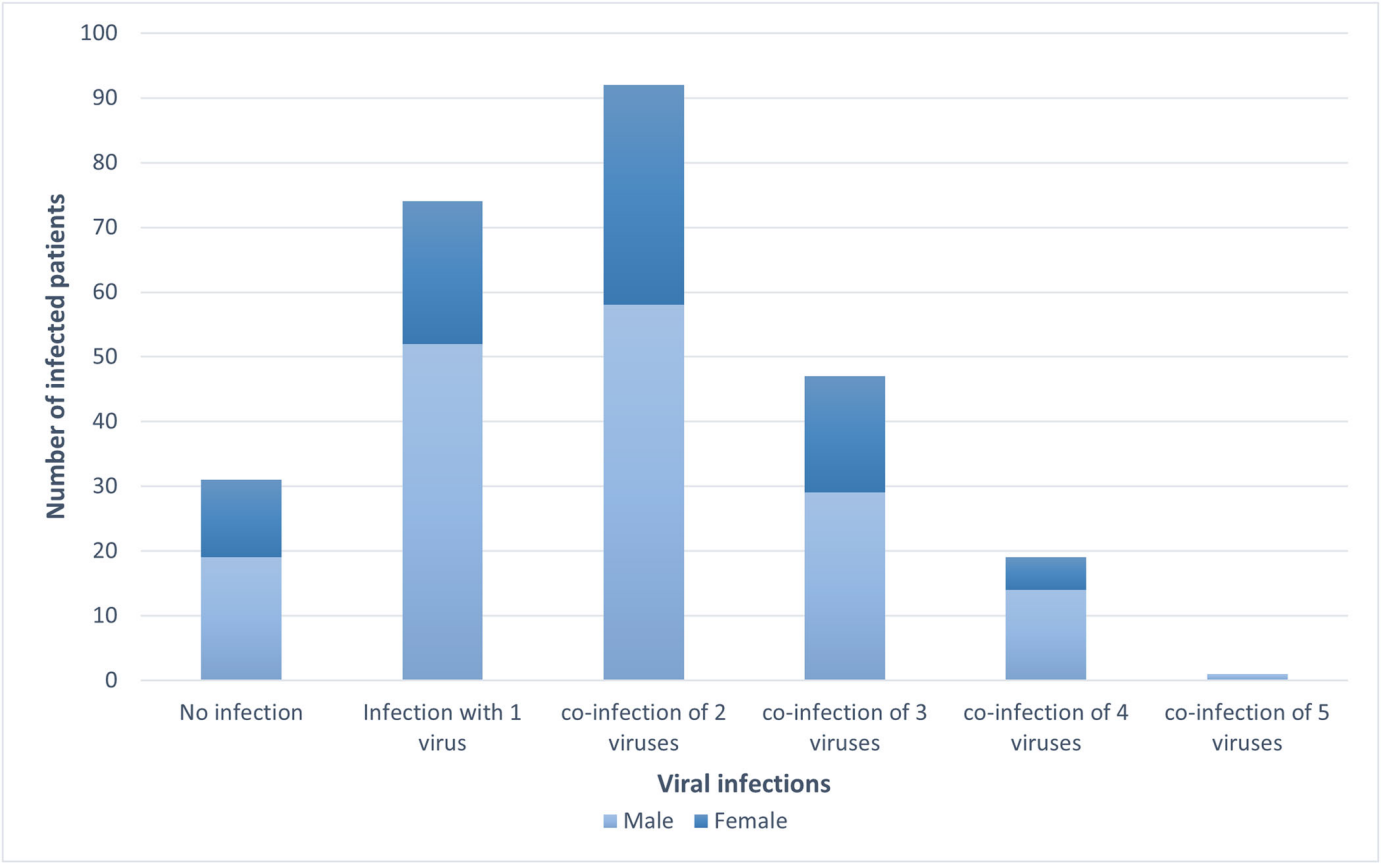
Frequency of different co‐infections by gender.

Among all cases, 159/264 (60.2%) had co‐infections, and 74/264 (28%) had a single virus infection. The male‐to‐female ratio was 1:0.51 among all infected patients. In the samples with single infections, the demographic distribution revealed a slight male predominance, with 52 males (70.3%) and 22 females (29.7%) affected. The cases with mixed infections exhibited (although nonsignificant) a more pronounced gender disparity, with 102 males (64.2%) and 57 females (35.8%) identified (Figure 3). Co‐infections were identified in a subset of cases, with various combinations. HPIV co‐infections were observed with HMPV, HRV, FLU‐A, RSV, and SARS‐CoV‐2. The co‐infection rates were as follows: HPIV‐1‐HPIV‐2 (0.37%), HPIV‐1‐HPIV‐3 (0.75%), HPIV‐1‐HMPV (5.7%), HPIV‐1‐HRV (5.7%), and HPIV‐1‐FLU‐A (0.8%). HPIV‐2 co‐infections were found with HMPV (6.8%), HRV (8.7%), and FLU‐A (0.4%). HPIV‐3 co‐infections occurred with HMPV (8%), HRV (3%), and FLU‐A (0.4%). Additionally, co‐infections involving HPIV‐4 were observed with HMPV (7.6%), HRV (4.2%), and FLU‐A (0.4%). RSV co‐infections were observed with HPIV‐2 (2.6%), HMPV (9.8%), and FLU‐A (0.4%). HRV co‐infections were found with SARS‐CoV‐2 (10.6%), RSV (9.8%), and FLU‐A (1.1%). The most frequently seen co‐infections were between HRV and HMPV (38%) and HMPV co‐infections with SARS‐CoV‐2 (7.2%), and FLU‐A (0.8%). Triple infections of HMPV‐HRV with RSV (6%), SARS‐CoV‐2 (5.3%), and FLU‐A (0.7%) were also identified. Significant associations were observed between co‐infections and increased rates of seizure (*p* = 0.01) and pneumonia (*p* = 0.03).

## 4 Discussion

ARIs represent a considerable health challenge for children worldwide, with implications for their families and societies, as well as significant health care system and economic burdens, especially in developing countries [16]. Respiratory viral infections, characterized by symptoms like fever, cough, and shortness of breath, contribute to high morbidity and mortality rates among children under 5 years old. Respiratory viruses are the most common pathogens in ARIs [17, 18], accounting for the majority of LRTI [19, 20, 21]. According to Esposito et al., infants in their first year of life are highly susceptible to respiratory infections [22]. Our findings revealed significant insights into the epidemiology of respiratory viruses in this population, highlighting key trends and variations in viral detection rates and clinical manifestations.

This study found that 88% (233/264) of children hospitalized for acute illness tested positive for at least one respiratory virus. Previous studies in Iran and other countries showed a rate between 59% and 95% for viral infection among hospitalized children [23, 24, 25]. One of the reasons for the high rate of respiratory infections in these patients could be that all of these children were hospitalized in a specialized hospital due to suspected viral respiratory illness. HMPV and HRV were the most frequently detected viruses in children hospitalized with ARIs during the study period. This is consistent with previous research indicating that HRV and HMPV are prevalent among children with ARIs [26, 27]. Our results showed a significant reduction in RSV and FLU‐A and B virus infections, compared to previous studies [26, 28]. For instance, studies conducted before the COVID‐19 pandemic reported higher prevalence rates of RSV, which was a major cause of ARIs in children [23, 27]. The observed changes in virus prevalence such as dominance of HMPV and HRV could be attributed to public health measures such as social distancing and mask‐wearing implemented during the COVID‐19 pandemic, which may have reduced transmission of certain viruses such as RSV and Influenza [17, 18]. Additionally, our findings support the notion that different respiratory viruses have varied seasonal patterns and are affected differently by public health interventions [24, 25]. For instance, in 2021 Kuang et al. investigated that the RSV levels were lower than expected following the emergence of SARS‐CoV‐2 and the implementation of pandemic mitigation measures. Interestingly, HMPV prevalence rose among pediatric patients, potentially due to asymptomatic or mild reinfections within households and communities, as well as extended viral shedding from recovering HMPV‐infected individuals [29].

The study found HPIV‐2 as the most prevalent type (12.9%), followed by HPIV‐1 (8.7%), HPIV‐3 (8%), and HPIV‐4 (7.6%). This is in accordance with a study by Ramezannia et al. in Tehran, Iran, where HPIV‐2 was the most common respiratory virus in pediatrics respiratory disease, followed by HPIV‐1 and HPIV‐4 [15]. Globally, across multiple countries, HPIV‐3 is often reported as the most prevalent, followed by HPIV‐1 and HPIV‐2 [4, 30]. The high prevalence of HPIV‐2 in our study suggests geographical or epidemiological variations, potentially due to different seasonal periods, demographic differences and the impact of COVID‐19 health measures, which may have changed the dynamics of viral transmission globally.

Age and gender may impact susceptibility to respiratory viruses [31, 32]. The observed gender disparity in positive cases, with males being more affected compared to females in both single (70.3% male vs. 29.7% female) and mixed infections (64.2% male vs. 35.8% female), aligns with existing research suggesting that males may have increased susceptibility to respiratory infections during early childhood [33]. Additionally, significant age‐related variations in virus detection were observed, with the majority of severe cases that needed hospitalization, occurring in the youngest children, particularly those under 2 years of age (65.6%). This increased susceptibility in younger children can be attributed to their immature immune systems and small diameter airways that get occluded if inflamed, therefore leading to severe respiratory distress, bronchiolitis, and pneumonia [34].

The severity of respiratory diseases varies among individuals due to several virus‐ and host‐associated factors [35]. The clinical presentation of respiratory viral infections in this study was consistent with previous reports, with common symptoms such as cough, fever, and shortness of breath being the most frequently observed [27]. However, more severe symptoms like cyanosis, wheezing, and pneumonia were strongly associated with certain viruses, particularly RSV, HRV, and HMPV. RSV was associated with severe respiratory conditions, including pneumonia and the need for mechanical ventilation, and is a leading cause of hospitalization in infants [36, 37]. Pneumonia was the main clinical diagnoses in HMPV infection similar to Wang et al. [38]. We observed other associations between HMPV airway infection and symptoms than previously reported, like HMPV infection associated with pneumonia and cough. This underscores the importance of early detection and management of HMPV infections to prevent life‐threatening complications.

Underlying diseases and preterm birth (before 37 weeks gestation) are known risk factors for severe respiratory infection in children [39]. Among the underlying disease, chronic heart disease was associated with HRV, suggesting that children with this condition may be more susceptible to severe outcomes of respiratory diseases. We did not find similar associations with neuromuscular disease and preterm birth, maybe due to a small sample size [40]. Variations in routine vaccination schedules and reduced exposure to respiratory viruses during the COVID‐19 pandemic may have impacted the population's immunity against respiratory viruses [41]. Despite the high vaccination coverage in the study population (86.7%), respiratory viral infections remained prevalent, particularly viruses like RSV, HRV, and HMPV.

In this study, 7.2% of the cases required hospitalization in the intensive care unit, and 1.9% (five cases) demised. No significant association was found between the severity of clinical symptoms and the poor outcome. All five deceased patients tested positive for at least one virus and HMPV proved positive in all cases. This is consistent with previous studies that emphasize the tentative severity of HMPV infections in pediatric populations [42, 43]. Furthermore, three out of the five deceased patients had underlying conditions, including kidney disease, neuromuscular disease, chronic heart disease, and Down's syndrome. Additionally, three of the deceased cases were coinfected with two or more viruses. It is worth mentioning that, given the small number of fatal cases in this study and the observational nature of the results, we cannot conclude that these deaths were caused by the viral infection.

The finding of a high prevalence of co‐infections, with 60.2% of the samples exhibiting mixed infections, is consistent with other studies in pediatric populations that report a significant occurrence of co‐infections, particularly among respiratory viruses [32, 44, 45]. This underscores the complexity of respiratory illnesses and highlights the importance of recognizing multiple pathogens in clinical settings as that may impact on outcomes. Among all types of co‐infections HRV and HMPV were the most frequently observed (38%), likely due to the widespread circulation of both viruses during the sampling period. The occurrence of respiratory viral co‐infections in the pre‐pandemic era varied from 31.2% to 61.8% in different regions [46, 47, 48]. During the pandemic, however, multiple studies observed a reduction in co‐infection rates, though this trend varied by geographical region and the specific viruses circulating in those areas, which may have impacted the study results [49, 50, 51]. While respiratory infections were anticipated to decline during the pandemic due to mask‐wearing and hygiene practices, the considerable arrival of tourists from diverse locations to the touristic city of Gorgan may be a contributing factor to the increased infection rate in the region.

This study demonstrated associations between co‐infections and clinical complications, such as seizures and pneumonia. The observed association between viral co‐infections and increased clinical complications in children may be explained by several immunological mechanisms. Co‐infection with multiple respiratory viruses can lead to dysregulated immune responses, including excessive cytokine release, impaired interferon signaling, and increased epithelial damage in the airways. Additionally, viral interference and competition for host immune resources may exacerbate respiratory symptoms and prolong disease course [52]. These results are consistent with other studies indicating that patients with co‐infections experience increased morbidity risk [53, 54]. Therefore, clinicians need to remain vigilant and aware of adverse effects due to the complexity of these co‐infections. This could complicate diagnostic and therapeutic approaches influencing treatment strategies and patient management. While supportive care remains the main treatment for most pediatric respiratory infections, the identification of multiple viral pathogens could help monitoring, especially in infants or those with underlying diseases [55]. Furthermore, in cases where specific antiviral therapies are available, timely and accurate viral diagnostics may help guide more targeted treatment decisions.

The rate of viral co‐infections is higher in children compared to other age groups [44]. Although some studies suggest no significant differences in clinical severity between patients admitted with single viral respiratory illnesses and those with co‐infections, preschool children with co‐infections appeared to be at a higher risk of death [56]. Among the five deceased cases of our study, three exhibited viral co‐infections involving triple and dual infections of HMPV with other respiratory viruses such as HPIV‐1, HPIV‐2, and HRV. These findings emphasize the need for physicians to be aware of the increased risk of co‐infections in children, as they can contribute to more severe disease progression. Early and precise diagnosis is crucial, as it allows for timely interventions that may reduce complications, especially in cases involving multiple viral infections.

Clinical management, vaccination strategies, and the surveillance of respiratory viruses play a crucial role in preventing respiratory diseases in pediatric populations [57, 58]. For instance, our data showed the dominance of HRV and HMPV infections among children. However, no preventive vaccines are currently available for these two viruses. This highlights the necessity for ongoing surveillance to monitor shifts in viral circulation and inform vaccination strategies. Rapid detection of respiratory viruses in children is critical. Our study revealed a high prevalence of co‐infections, emphasizing the need for molecular diagnostic techniques capable of rapidly detecting these viruses with high sensitivity and specificity [59].

Several limitations should be taken into account in this study. First, this was a hospital‐based epidemiological study with a limited sample size, conducted at a single tertiary pediatric center in Golestan Province, Iran. In other regions or hospitals, the patterns of viral spread, along with associated clinical symptoms and outcomes, may vary, meaning that the results may not fully reflect the burden of respiratory viruses in the broader community. Moreover, multicenter studies are necessary to increase the sample size and address this key limitation. Second, in this study, we just examined the frequency of viruses in hospitalized individuals; further examination of respiratory viruses in nonhospitalized children should be considered. Third, all our respiratory samples were collected during a short period in the winter season, which may have influenced the overall frequency of these viruses. For example, the absence of FLU‐B, besides the public health measures, could be linked to local transmission dynamics or the relatively narrow time frame of sample collection. Furthermore, these findings may be season‐dependent and might not necessarily apply to other seasons or years. Fourth, while a high rate of viral co‐infection was observed, we did not perform sequencing or apply quantitative viral load measurements to distinguish active co‐infections, which may lead to overestimation of co‐detections so these findings should be interpreted with caution. Lastly, this study examined a wide range of respiratory viruses in children, and only basic descriptive and analytical analysis of their frequencies was done. More comprehensive analyses of epidemiological characteristics, risk factors, co‐infections, and disease severity of these viral pathogens need to be performed. Additionally, potential confounding factors such as comorbidities or prematurity were not controlled for, which may influence the interpretation of clinical outcomes.

## 5 Conclusion

This study reveals a significant burden of ARIs in children under five in Golestan Province, Iran, during the COVID‐19 pandemic, with HRV, HMPV, and RSV being the most common viruses. This study emphasizes the need for early detection and management of multiple viral infections in children, calling for continued surveillance and broader studies to understand the impact of these infections, especially in high‐risk groups and different regions.

## Author Contributions

**Ali Nateghi:** data curation, investigation, methodology, project administration, writing – original draft. **Morvarid Hamrahjoo:** data curation, investigation, methodology, project administration, writing – original draft. **Mohammad Yasaghi:** data curation, formal analysis, investigation, methodology, software. **Mahnaz Ramzali:** data curation, investigation, writing – review and editing. **Saeed Samadizadeh:** validation, writing – review and editing. **Fatemeh Fotouhi:** validation, writing – review and editing. **Vahid Salimi:** validation, writing – review and editing. **Lobat Shahkar:** validation, writing – review andediting. **Britt Nakstad:** validation, writing – review and editing. **Alireza Tahamtan:** conceptualization, methodology, project administration, supervision, visualization, writing – review and editing.

## Consent

The parents of all participants were informed about the purpose of the study, and their informed consent was obtained.

## Conflicts of Interest

The authors declare no conflicts of interest.

## Supporting information


**Supplementary Table 1:** Selected primers for amplification of viruses.

## Data Availability

The authors confirm that the data supporting the findings of this study are available within the article. In addition, anonymized datasets can be made available upon reasonable request to the corresponding author. All authors have read and approved the final version of the manuscript, had full access to all of the data in this study and take complete responsibility for the integrity of the data and the accuracy of the data analysis.
