# Supplementary material for: Epidemiology and Clinical Features of Respiratory Viruses in Hospitalized Iranian Children During the COVID‐19 Pandemic
Source: Immun Inflamm Dis. 2025 Sep 29;13(9):e70275. doi: 10.1002/iid3.70275 (PMC12477401; doi:10.1002/iid3.70275)
Supplement: Supplementary file 1 — Supplementary Table 1: Selected primers for amplification of viruses. [file IID3-13-e70275-s001.docx]

**Supplementary Table 1**. Selected primers for amplification of viruses. Degenerate bases are represented as follows: K = G or T, M = A or C, Y = C or T, R = A or G.

| **Virus** | **Gene** | **Primer** | **Primer length (nt)** | **Product TM (**°**C)** | **Product length (bp)** |
| --- | --- | --- | --- | --- | --- |
| **HPIV-1** | HN | F: 5’- ACATCKCGTGTTAATCCTACCAT - 3’ | 23 | 77 | 94 |
|  |  | R: 5’- TGTATGCTGCKTCTAATTRTCCA -3’ | 23 |  |  |
| **HPIV-2** | HN | F: 5’- ATGCCACMACTGAKCTAGCTGA -3’ | 22 | 80 | 139 |
|  |  | R: 5’- TGATAGATCCCGCTTCCKACT – 3’ | 21 |  |  |
| **HPIV-3** | HN | F: 5’ATATACTGATGCATATCCKCTCA – 3’ | 23 | 84 | 175 |
|  |  | R: 5’- GCAGCTMGTTGTTGTATAKCCA – 3’ | 22 |  |  |
| **HPIV-4** | HN | F: 5’- GTCAACACCAGGGAATTTGC -3’ | 20 | 73 | 70 |
|  |  | R: 5’- ATTCTCCATAAGTTCATTGAGC - 3’ | 22 |  |  |
| **FLU-A** | M | F: 5’- GACCRATCCTGTCACCTCTGAC- 3’ | 22 | 81 | 106 |
|  |  | R: 5’- AGGGCATTYTGGACAAAKCGTCTA -3’ | 24 |  |  |
| **FLU-B** | M | F: 5’- CACTTTCTTAAAATGTCGCTGT -3’ | 22 | 86 | 125 |
|  |  | R: 5’- TCAAATTCTTTCCCACCGAAC - 3’ | 21 |  |  |
| **HMPV** | M | F: 5’- TATGGCAAAGCATTAGGCTCA - 3’ | 21 | 84 | 90 |
|  |  | R: 5’- TTGACCAGCACCATAAGCTTG - 3’ | 21 |  |  |
| **HRV** | 5'-NC region | F: 5’- AAACAACAGATACCGTTATCCG-3’ | 22 | 93 | 115 |
|  |  | R: 5’- CAGCCTCATCTGCAAGGTC - 3’ | 19 |  |  |

HN: Hemagglutinin-Neuraminidase, M: Matrix, 5'-NC region: 5'-Noncoding region, F: Forward primer, R: Reverse primer, nt: Nucleotide, bp: Base pair,
